# Effects of coastal protection structures in controlling erosion and livelihoods

**DOI:** 10.1016/j.heliyon.2023.e20633

**Published:** 2023-10-04

**Authors:** Bapentire Donatus Angnuureng, Richard Adade, Ernest Obeng Chuku, Selorm Dzantor, Emmanuel Kwadzo Brempong, Precious Agbeko Dzorgbe Mattah

**Affiliations:** a Africa Centre of Excellence in Coastal Resilience, Centre for Coastal Management, SBS, University of Cape Coast, Cape Coast, Ghana; b Department of Fisheries and Aquatic Sciences, School of Biological Sciences, University of Cape Coast, Cape Coast, Ghana; c LEGOS (University of Toulouse/CNRS/IRD/CNES), Toulouse, France

**Keywords:** Coastal protection, Vulnerability index, Livelihood, Erosion, Nature-based solutions

## Abstract

The fiscal and social cost of ameliorating the impact of coastal erosion resulting from climate change is an increasing burden for coastal states, and in developing nations the physical interventions implemented may present a double agony – increasing debt levels and potentially obstructing livelihoods in the rural coasts. Against this background, this study was conducted to explore the impact of hard-engineered coastal protection on coastal vulnerability and community livelihoods in Ghana using a combination of Unmanned Aerial Vehicles (UAV), geographic information system tools and social survey. Shoreline change analysis by the application of the Digital Shoreline Analysis System (DSAS) with aerial photographs from 2005 to 2022 reveals an average statistical rate of change of −1 m/year in shoreline erosion of the beaches. A computation of coastal vulnerability indices for fourteen beaches, incorporating coastal protection as an additional parameter shows that from east to west, hard-engineered coastal protection structures slowed the rate of erosion, whereas unprotected beaches have highly eroded, stressing the importance of coastal protection. In consequence, coastal protection has dire livelihood-reduction implications for coastal inhabitants who are predominantly artisanal fishers. A lack of acceptable consultation with the communities exacerbates the effects from these hard-engineering interventions. The beaches of high vulnerability concerns are Dzita, Ada, Sakumono, Glefe, Apam, Anlo, and Busua. To safeguard the livelihoods of vulnerable coastal communities, we support a shift from hard engineering to more integrated and nature-based coastal management approaches on a national scale since most parts of the coast are now susceptible to erosion in contrast to what was previously observed that only the eastern part of the coast was highly vulnerable.

## Highlights

• Coastal vulnerability is spread along the entire coastline of Ghana.
• Grey coastal protection decreases livelihood opportunities for coastal communities.
• A lack of acceptable consultation with coastal communities exacerbates the effects from these hard-engineering interventions.
• Examination of the vulnerability shows beaches along hard-engineered coastal protection in Ghana are not absolutely protected.
• The application of nature-based solutions in Ghana and many other coastal countries is low.

## 1 Introduction

Escalating climate change that threatens coastal cities and increases vulnerability and adaptation challenges could intensify future coastal erosion and flooding [1,2]. The impact of wave induced currents and wave energy on coastal erosion is exacerbated by storms or tidal surges, which are of larger energies albeit short time, and thus crash fiercely onto shore. The resulting erosion of beaches and shorelines has negatively affected the lives of people living along the coast and caused extensive damage to their properties and public infrastructure to date [2]. Nonetheless, efforts at quantifying the erosion hazard have been challenging particularly at engineered areas where the natural equilibrium of the beach has been changed. In most places, this requires monitoring programs covering an entire seasonal cycle [3].

On many shores impacted by erosion, interventions have included the introduction of shore structures such as groynes, jetties, breakwaters, and revetments (e.g., [4]; [5]; [6]; [7]), but after extensive review [8], it is believed that these structures transfer the problem to adjacent beaches. They obstruct the parallel sediment transport [9,10]), leading to sediment imbalance, updrift deposition and downdrift erosion. While coastal protection structures help slow erosion caused by wave-induced forces, some of these structures prevent the shifting of sand along the beach, thus, are themselves problematic [10] as these structures influence significant erosion adjacent them. Hard interventions such as coastal protection structures may not fully be effective or cost-effective since their construction is costly [11].

Aside erosion, literature has shown that the construction of these hard erosion control structures results in a decrease, and permanent loss in some cases, of living resources along affected shorelines [12]. In most cases, both fauna and flora, as well as economic activities of coastal communities could be seriously affected rendering the communities poorer than they were. Coastal development and the construction of structures as well as natural phenomena (e.g., monsoon currents, storm surges, and tides) have resulted in the disappearance of specific beach areas that were previously used for canoe docking [13]. In their book ‘The Last Beach,’ Pilkey and Cooper [14] emphasize that sea walls, often considered protective measures against erosion and rising sea levels, actually contribute to beach destruction and require costly and frequent reconstruction. As a result, the challenges faced by businesses, farmers, artisanal fishers and other members of fishing communities are exacerbated. These observations indicate that current approaches to managing coastal erosion have not reached their full potential [14,15]. Despite the inherent challenges associated with the natural evolution of beaches, the construction of coastal protections continues along Ghana's entire coastline without proper planning and in a disorganized manner.

Along the coastline of Ghana, grey infrastructures implemented to defend the coastline cover roughly 15% of the 550 km coastline, yet coastal erosion is persistent and spreading along the rest of the coastline. The developments of structures are often required to be followed by rigorous forms of environmental and social impact analysis (ESIA) or scientific assessments of the socio-economic impact aftermath. As far as it is known, the vulnerability of beaches where grey infrastructures are implemented along the coastline of Ghana, their performance has not been assessed although it has long time been known that coastal protections are themselves parameters that affect the beach stability. Coastal protection structures as an anthropogenic factor equally influences the vulnerability of coastal communities. Including the presence of these coastal protections as a parameter in the computation of the coastal vulnerability index could reveal their potential impacts on the resilience of the beach. Vulnerability of beaches to coastal hazard such as erosion and sea level rise is defined here as how exposed and sensitive a particular location can be as well as the impact and its ability to adjust to these impacts [16]. Coastal vulnerability assessments have mainly focused on beaches without any coastal defenses, leading to limited knowledge about how structures like groynes, jetties, revetments, or breakwaters affect beach dynamics. The impacts of these defenses on beach changes are still not well understood. However, conducting a Coastal Vulnerability Index (CVI) analysis on protected areas provides a valuable opportunity to visually assess and prioritize the effectiveness of different coastline management strategies.

The main objective of this paper is therefore to assess the transitional vulnerability indices of defended beaches as a consequence of existing hydro-morphodynamic features and their associated socio-economic implications along the coast of Ghana. This study assessed the CVI of selected dynamic beaches along the coast of Ghana using a modified USGS approach with sea defense parameter. The findings from this study should assist in the refocusing of policy on how coastal protections are constructed and oriented within fishing communities and in addition towards efficient protection of the beaches.

## 2 Methods

### 2.1 Description of the coastline of Ghana

**Fig. 1:**
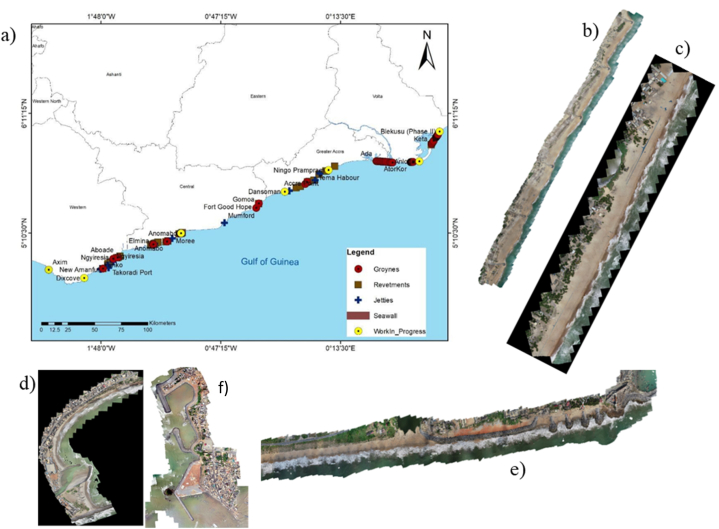
a) The map of Ghana showing different areas with coastal protections and those under construction and Orthophotos of beaches and coastal protections at b) Keta beach with groynes c) unprotected beach at Dzita d) Elmina e) Cape Coast f) Axim.

The Ghanaian coast (Fig. 1) is one of the longest in West Africa, covering about 550 km. This coast provides a home for many highly specialised fauna and flora [17]. The coastal environment of Ghana is characterised by the presence of sand and rocks [18,19]. On the east, where the coast is predominantly sandy and low-lying, the beach is highly vulnerable to episodic erosion and coastal flooding from wave overtopping. From the Central to the West, where there are highlands and sand interspersed with rocks, erosion, and flooding are minimal. At the various areas where erosion is pronounced, there have been some interventions as indicated in Fig. 1a, in the form of hard coastal protections [8], to protect properties and lives. Fig. 1b, d, 1e, and 1f, represent groynes, jetties, and revetment types of interventions commonly seen along the entire coastline of Ghana. However, beaches like Dzita (Fig. 1c) are unprotected, so provide sandy and recreational sites. Along the coastline, there are various communities involved in fishing for their livelihood through the use of man-powered boats and boats with outboard motors. Introduction of new structures therefore can change their activities. The hydrodynamic conditions including waves, tides and currents along the coastline are moderate with significant wave heights at most 3 m, wave peak periods ranging between 6 and 20 s and while tidal range is around 1 m. The entire beach has a mean longshore drift from west to east following the Guinea current.

### 2.2 Beaches comprising coastal protections

Data was collected on fourteen selected beaches (ten defended beaches and four undefended beaches) with unique characteristics for geomorphological and socio-economic evaluation. Consideration of the beaches was based on the presence of coastal protection, reported erosion rates, fishing activities, and data availability.

Fig. 1b is the beach of Keta between Kedzi and Woe. The monitored part is about 4 km long beach near the Keta Lagoon complex. The Keta beach is bordered by swampy areas and a large sandspit (a sandspit is a deposition bar or beach landform off the coasts) along, barely wider than 2.5 km at its widest point with an elevation up to 2 m above mean sea level [[20], [21], [22]]. This beach was eroding significantly before the year 2000, so a sea defense was implemented between 2000 and 2004. The Keta Sea Defense project was the first existing coastal protections in Ghana. This involved six groyne structures, positioned at intervals of around 700 m with 2.6 million m^3^ of beachfill (D_50_ ∼ 0.6 mm) to protect a 5 km rapidly eroding shoreline [23]. The Project also included the construction of an 8.5 km highway link using dredged sand, and 200 ha of land reclamation. Over 12 million m^3^ of sand and unsuitable sediment was dredged and over 1 million tons of rock placed in the project [23].

Other engineered beaches that were investigated in this study include the Ada, Sakumono, Glefe, Mumford, Anomabo, Cape Coast, Elmina and Axim beaches. Since the damming across the Volta River in 1964, coastal erosion increased significantly [21,24] at Ada, even at locations upstream. Yearly shoreline recession became 6 m/year. A coastal protection comprising of 7 groyne structures and beach nourishment was designed and implemented between 2012 and 2015 at Ada with the aim to control the erosion.

Sakumono beach on the other hand was experiencing alarming rate of erosion, so a seawall and two (2) km revetment were constructed on that coastal stretch [25]. Glefe beach is comparatively an open low-lying coastline facing strong continuous swell waves [26,27] which breaks obliquely as they enter the beach area, leading to erosive longshore currents. Glefe beach undergoes high recession. Coastal protection by revetments were done particularly to protect the salt production area against erosion.

A modern fish landing port including jetties were constructed at Mumford in 2021. At Anomabo, a long revetment was constructed to protect the beach. At Cape Coast, a revetment and four riprap groynes were constructed in 2020 (Fig. 1e) on the western side of the Cape Coast castle to protect the beach. To minimize the erosion of the Elmina beach (Fig. 1d), two jetties were constructed in 2007–2011 and revetment in 2017 to protect the Castle and nearby community but that as well did stop the erosion. Currently, the entire beach is fenced with boulders in the form of revetment. Axim beach which is in the western region of Ghana is a mixture sand and rock coastal area with features such as bays, islands and headlands. To address erosion problems, sea defence walls were built in the 1960s, initially effective but later deteriorating and leading to severe erosion. Unfortunately, poor engineering decisions and siting amplified the impact of waves and erosion, creating new risks for the fish landing site and posing challenges for Axim's coastal community. Subsequently, the government commenced coastal protection which was completed in 2020.

The main aim of these projects is to erect a protective barrier to combat the detrimental effects of erosive forces. These interventions for stability and reduction in vulnerability of the beach along this area are assessed in this paper to determine their efficiency. The key informants of these communities were then interviewed, and the erosion status of the beach was evaluated. The main features of the various coastal protections presented above have been summarized in Table 1.

**Table 1:** Coastal protections, purpose, completion date, and types of structure and lengths.

Projects	Reason for Protection	Date completed	Type of Structure/Stretch for protection
Keta Coastal Protection Project	Protection of Keta Township from sea flooding.	2004	Armour rock groynes/revetment/beach/nourishment.
Ada Coastal Works Phase 1 &2	Protection of Ada Township/Promotion of Tourism/Enhancement of Salt Industry.	2016	Armour rock groynes/beach nourishment on a total stretch of 16.6 km.
Sakumono Coastal Protection Project. Phase 1& 2	Protection of Railway Line linking the national capital Accra to the major industrial city of Tema.	2016	Armour rock revetment/groynes at a stretch of 5 km.
Anomabu Coastal Protection project	Protection of Anomabu Fort and the Anomabu township.	2021	Armour rock revetment/groynes at a stretch of 5 km.
Cape-Coast Coastal protection Project	Protection to the Cape Coast Castle and Cape-Coast Township.	2020	Armour rock revetment/groynes at a stretch of 5 km.
Elmina	Protection to the Elmina Castle and Township.	2017	Two jetties, and revetment.
Dixcove Coastal protection Project	Protection of Dixcove Township and Enhancing the fishing industry.	2017	Armour rock revetment with beach stabilization for a coastal stretch of 2.4 km.
Glefe	Protection of Glefe and Wiabom township	2019	Revetments
Axim Coastal Protection Project	Protection of Axim Township/Promotion of Tourism/Enhancing fishing.	2021	Armour rock groynes/Jetties/breakwater on a stretch of 5 km

### 2.3 Beaches without coastal protection on them

Three beaches which were not protected were also evaluated. These include Dzita beach (Fig. 1c), Apam, and Busua beaches. These are presented here as non-defended shorelines to compare their vulnerability with those that are defended. Dzita beach has been monitored by video camera installation [22] and unmanned aerial vehicle (UAV) to understand its morphodynamics and changes in sediment budgets [22,28]. Large beach changes have been observed and related to relatively high wave events. Shoreline changes showed erosion at rates of −7.23 ± 0.23 to – 4.85 ± 0.23 m/yr in 2019. In 2018–2019 alone, this beach lost on average 7 m. At this speed, key coastal infrastructure and resources are prone to erosion hazards.

Apam beach that is considered in this current study is sandy, with approximately 1.9 km in length. It is arguably one of the busiest fish-landing sites along the coastline [29,30]. Sediment sizes range from 0.90 mm to 2.10 mm [30]. Anlo Beach was chosen because it is among the beaches in Ghana with high reports of flooding and erosion making the coastline stability very challenging. The sediment discharge trends from the Pra river, and particle size distribution does not fully support beach stability [31]. Frimpomaa [31] revealed that the average discharge for the Pra River was computed to be 188 m^3^/s, and 4537.03 tons/day. Out of this, 52% is made of very fine bedload (with sizes less than 0.062 μm), which makes it plausible to infer that the sediment supplied would not be able to fully support beach building. It is therefore essential to assess how vulnerable this beach could be, compared to the different types of beaches.

Busua is a coastal town situated in Western Ghana. The beach of Busua under study is 1.7 km featured between rock outcrops and drained by a stream. It is a well-known recreation and relaxation hub as it hosts a string of resorts, restaurants and hotels. It experiences a good degree of erosion because the littoral drift is insignificant due to the restrictions from the headlands [32].

### 2.4 Morphological data collection

The study sought to analyse the effectiveness of the protection given by the hard structure over the period 2005 to 2022 using high resolution aerial images from aerial photography and drones with DJI Phantom 4 pro. In addition, we gathered geomorphological parameters and hydrodynamic conditions to evaluate the vulnerability of these beaches and the selected undefended shores within the same period, with changes in the undefended shores as a control group.

#### 2.4.1 Shoreline surveys by DJI Phantom 4 Pro UAV (drone)

One time beach survey was carried out on each of the fourteen beaches described above by drone flights between January 2022 and March 2022. Drone flights were conduct at an altitude of 100 m. At least 300 images were collected at all sites. We filtered collected images by removing blurry images. Multi-View Stereo and structure from motion algorithms were employed for three-dimensional image reconstruction [33]. First, images were aligned [34], secondly, reconstruction of pixel-based dense stereo was used to line up data and thirdly, an orthophoto was built and used to develop a digital elevation model (DEM). The DEM was classified highly at areas where fishing boats, sea defenses and houses were recorded on images, for the isolation of the exposed ground. This help to minimize the errors in topographic heights. Total beach elevation errors (Z) and shoreline positional errors (XY) from UAV images were 0.6 and 7.0 cm, respectively. Orthophotos were uploaded into ArcMap where shoreline positions were manually digitized with the high-water mark proxy [35] in consistence with the proxies used for shoreline identification in Ghana [22,36].

#### 2.4.2 Historical shoreline position and shoreline rates of change

To estimate the shoreline change rates at each of the sites, 2005 shoreline of the coast of Ghana was used in combination with the shorelines extracted from the UAV images. The 2005 shoreline was extracted from an aerial image of the entire coast obtained from the survey department of Ghana and the shoreline digitized using the High-Water Mark (HWM) as the proxy. The onscreen digitizing method of mapping the shoreline proxy was used to extract shoreline positions from the aerial photo. The total error associated to the 2005 shoreline extraction was 4.5 m due to HWM proxy used and the precision in digitization.

The shorelines were analysed in ArcMap using the digital shoreline analysis system (DSAS) [37]. The spatial and temporal shoreline migration [38] were calculated in the DSAS. This software is based on three components of baseline, shorelines, and transects. Following Weerasingha and Ratnayake [38] and Thieler et al. [37], a baseline was first created seaward. After that, transects were generated perpendicular to the reference baseline with 5–15 m spacing. The investigated beaches were of different lengths, therefore we used different intervals to have efficient processing. The endpoint rate (EPR) approach of DSAS was applied for the calculation of shoreline changes. EPR is applicable when only two period data is available, in this case 2005 and 2022. The endpoint rate (EPR) is calculated as:

(1) $$\text{EPR}=\left(\frac{net\;shoreline\;movement\;in\;distance}{time\;between\;oldest\;and\;youngest\;shoreline}\right)$$

### 2.5 Computing the coastal vulnerability index (CVI)

The vulnerability of the selected beaches is shown via the exposition of these beach regions to climate factors like sea level rise, the sensitivity to a specific coastal system, and the adaptive capacity of the coastal system to changes. The vulnerability was estimated by computing an index based on seven physical parameters given in Table 2 by using the relation in Equation (2). The CVI here is that used in Thieler and Hammar-Klose [39] and Gornitz et al. [40], and the sensitivity index employed by Shaw et al. [41]. The following variables were used; (a) average absolute sea level rise (SLR) rate (mm/yr), (b) coastal slope (%), (c) shoreline change rate (m/yr) (d) lithology and geomorphology of coastal landforms (based on the relative resistance of a given landform to erosion), e) *Hs* (m), (f) *TR* (m), and g) coastal protection. Shoreline change (*<X>*) was obtained from the EPR results between 2005 and 2022 as explained earlier. Nearshore slopes were computed using global interpolated MERIT + GEBCO merged bathymetric dataset [42] of the coastline with transects perpendicular to the Open Street Map coastline at every 1 km in the alongshore direction. The slope profiles were calculated as the ratio of the depth of closure to the horizontal length of the nearshore using these topo-bathymetric datasets that were created [42]. Significant wave heights (*Hs*) were averaged from ERA5 hindcast data [43] between 2005 and 2022, while tide range was extracted from WX Tide model [44]. Each of the used parameters had been addressed to the vulnerability rank (1–5) following the USGS approach (Table 2).

**Table 2:** Ranking of parameters following USGS CVI calculation.

Rating	Very low	Low	Moderate	High	Very high
*score*	1	2	3	4	5
Shoreline changes (m)	>2.0	1.0–2.0	−1.0 to 1.0	−2.0 to −1.0	<-2.0
Slope (%)	>0.2	0.2–0.07	0.07–0.04	0.04–0.025	<0.025
Geormophology	Rocky cliffed	Medium cliffs	Low cliffs	Cobble beaches, estuary, lagoons	Barrier and sandy beaches, Deltas, salt marsh
Mean Hs (m)	<0.5	0.5–0.85	0.85–1.05	1.05–1.25	>1.25
SLR (m/yr)	<1.8	1.8–2.5	2.5–2.95	2.95–3.16	>3.16
Tide range	>6.0	4.1–6.0	2.0–4.0	1.0–1.9	<1.0
Type of coastal protection	Sea walls	Revetments	Groynes, jetties, break waters	Sandbags, wood, and grass	No Defense

The USGS CVI consisted of only six parameters, however, in this paper, we have added the presence of coastal protection as a parameter. Based on the difference in efficiency and responses by coastal protections structures as reported in previous studies [8,45], the coastal protections were ranked accordingly. For instance, sea walls completely prevent any form of erosion, thus, providing very low vulnerability whereas beaches protected by sandbag structures, wood or grass may still experience some form of erosion. This method produces numerical data that cannot be equated directly with physical effects, but it emphasizes areas where the various effects of sea-level rise may be the greatest.

The data in Table 2 and the ratings given correspond to results on the field along the coastline of Ghana. In Ghana, average erosion is less than 2 m/yr, so when erosion is more than 2 m per year, then the beach is highly vulnerable. Even though parameters like tides, waves, and sea levels have not been measured at local scale for most of the beaches studied here, the classification given in Table is within reported regional values.

The highest and lowest values of the CVI were determined from Eq. (2).

(2) $$\text{CVI}=\sqrt{\frac{\left(a*b*c*d*e*f*g\right)}{7}}$$

where a, b, c, d, e, f, and g are the parameters in Table 2.

### 2.6 Determination of communities’ perspectives on the impact of coastal protection structures

Mainly qualitative data was collected from the coastal communities. Techniques used were mainly key informant interviews and observations. The essence is to examine the impact of the coastal protection structures on the environment and socio-economic conditions of the communities. This is important to exploring the extent to which the structures were beneficial to the communities. The key informants were purposively selected as a result of the important roles they play in community development. This study defined key informants as opinion leaders who had lived continuously in the communities for 30 years or more. A key informant interview guide was developed and used in eliciting information on changes observed in the communities by the respondents, factors of change, coastal features at risks, livelihood at stake, property lost, benefits and environmental challenges posed by the defense structures. The interviews were conducted in the local languages of Fante, Ga and Ewe, from west to east of the country. Overall, 30 key informants were interviewed with three (3) from each study site. With approval of the key informants, the interviews were recorded so as to capture correctly their submissions and to reduce the possibility of compromising the details of information provided by each of them. The recorded interviews were transcribed and analysed manually after copious reading to derive themes from the responses.

Observations were done by the research team on state of the environment at the time of data collection. Some of the key things observed included signs of continuous erosion, signs of sediment deposition, the manner of fishing as necessitated by the installed defense system, how and where communities berth their fishing canoes, presence of some biodiversity such as ghost crabs etc. Where necessary, questions were asked the key informants to ascertain the observations made.

## 3 Results

The results presented include the shoreline changes (retreat or advance), coastal vulnerability index (CVI) and the responses by community members on the import of coastal protections. A summary of the shoreline changes along the various beaches that were computed by the EPR approach is provided in Table 3. The mean is the alongshore-averaged cross-shoreline changes at all transect lines between 2005 and 2022.

**Table 3:** Shoreline change rates between 2005 and 2022.

Beach	Maximum (m/yr)	Mean (m/yr)	Minimum (m/yr)
Defended beaches			
Keta	4.7	1.7	−2.6
Ada	4.2	−0.1	−4.7
Sakumono site 2 (east)	0.9	0.2	−0.7
Sakumono site 1(west)	-(43.2)	-(33.6)	-(27.0)
Glefe	2.8	1.7	−0.2
Mumford	5.5	0.8	−1.9
Anomabo-Egyaa	4.1	1.3	−1.2
Cape Coast	5.0	1.2	−0.1
Elmina	2.9	1.0	−2.3
Dixcove	6.7	2.8	0.0
Axim	4.1	0.4	−0.9
**Unprotected beaches**			
Dzita	-(3.1)	−2.2	-(0.8)
Apam	0.9	−0.7	−4.5
Anlo Beach	1.6	−0.7	−2.5
Busua	2.3	0.0	−2.5

### 3.1 Shoreline changes along the coastlines

**Fig. 2:**
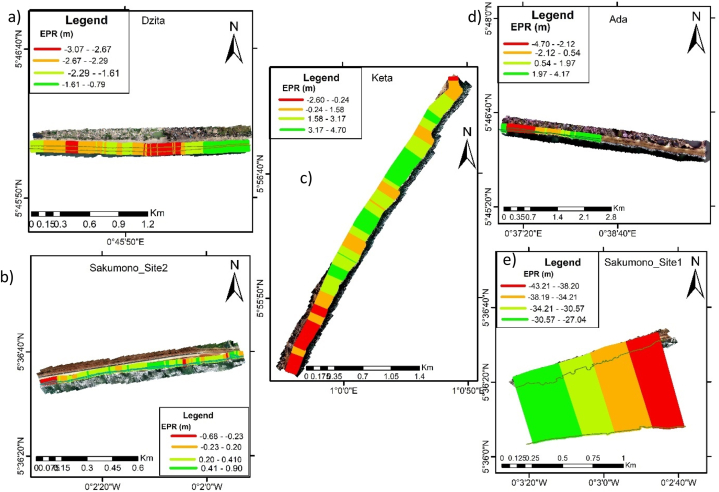
Shoreline changes along the eastern coast for a) Dzita Beach, b) Sakumono West Beach c) Keta Beach d) Ada Beach and Sakumono East Beach.

Shoreline changes between 2005 and 2022 were extracted from ArcGIS following the EPR method of DSAS for all the beaches that were studied. Fig. 2 presents the shoreline changes of the beaches in the Eastern corridor of Ghana's coastline: Dzita, Keta, Ada, and Sakumono beaches.

Keta Beach: This beach was one of the first to be engineered along the entire Ghanaian coast in the 2000s. The studied area covers only the part where 6 groynes of about 2 km was done. The EPR results for Keta indicate that most parts of the defended side are stable or building. Nonetheless, erosion is still observed outside where these groynes covered, particularly at the extreme updrift and downdrift ends of the coastline.

Dzita beach: This beach is one of the undefended beaches extending between Atorkor and Ada. The EPR results show that the entire beach is retreating. In 2020, Angnuureng et al. [22] measured a recession rate of 7 m/year, which suggest an estimated area of 200 m of beach could be eroded in a space of 30 years. Table 3 provides the minimum, mean, and maximum erosion rates of change as −0.8, −2.2 and −3.1 m/yr between 2005 and 2022. This means in recent years, Dzita beach has been eroding larger than the last two decades.

Ada Beach: Ada beach is a site of significant interest because the mouth of the Volta River is very dynamic. Shoreline changes at this beach have been estimated by our approach to exceed 4 m/yr in erosion and accretion (Fig. 2, Table 3). Long term community dwellers have indicated that erosion around this beach became worse ever since the sea defense was started 2012 and wave overtopping has increased.

Sakumono Beach**:** This beach is located about 200 m at the western side of the Tema harbour, the largest harbour in Ghana. Our observations mean that the immediate west of the harbour experiences minimal changes in terms of erosion compared to when you move further west. Close to the habour, the shoreline changes are not high, rates are less than 1 m/yr for both retreat and advance. However, further west, there is observed severe erosion on an average of −33 m/yr. Between 2005 and 2022, this beach has retreated inland by about 1 km.

**Fig. 3:**
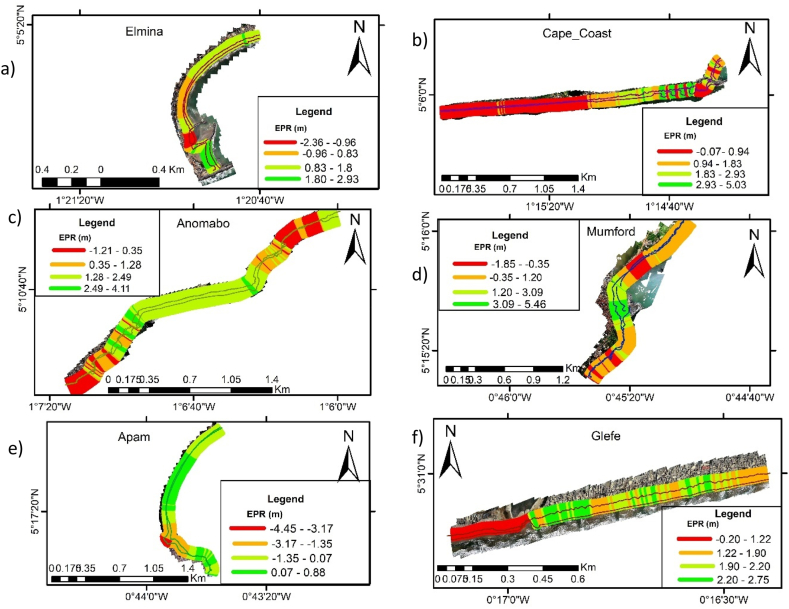
Shoreline changes along the central coastline of Ghana a) Elmina Beach; b) Cape Coast Beach c) Annomabo Beach; d) Mumford Beach; e) Apam Beach f) Glefe Beach.

Elmina Beach: Fig. 3a is the part of the Elmina coastal area that was investigated. It is of bay nature. This is a beach that has been engineered several times. The first was a sea wall that got destroyed before the jetties and revetments. Currently the entire beach is fenced with a revetment and a mini port is being built. Previous studies have noted that this beach was eroding around 3.4 m/yr between 1895 and 2002 [32], and later accreting around 4 m/yr between 2019 and 2022 [19]. This present study found that between 2005 and 2022, the shoreline is advancing at 1 m/yr (Table 3, Fig. 3a). The accretion here is believed to be due to the various interventions since 2012.

Cape Coast Beach**:** This area includes the beach surrounding the Cape Coast castle about 3 km length (Fig. 3b). About 95% of this beach is not eroding. A major part of the beach is building. One important observation on this beach is the fact that erosion is happening between groynes. On this beach, one will notice that close to the castle to east, where the groynes are located, it is green (accretion) and shows red (erosion) between the spaces. Erosion rates are however still very small in absolute magnitude, though our field observations suggest erosion between the groynes will worsen. This erosion between the groynes is because sand seems to be transported offshore more than what is received as the groynes are too high causing wave reflection and resulting in local scouring. These perpendicular groynes do allow sand from updrift coast to accumulate between the groynes.

Anomabo-Egyaa Beach: The results obtained by applying DSAS EPR to the beach indicates that 10% of the beach is eroding though at low rates while 90% is either stable or accreting. Averagely, the whole beach is accreting at a rate of 1.2 m/yr (Table 3). It was noticed that erosion of this beach is mostly observed at places (Fig. 3c) where the coastal protection does not cover.

Mumford Beach**:** Mumford beach (Fig. 3d) is surrounded by high hills and high vegetation. This means that the community is free from coastal flooding in the current years. However, coastal erosion had been observed which led to the construction of jetties and mini port for the fishing community. This study finds that about 25% of the beach is eroding up to about 2 m/yr, while the rest of the 75% is either stable or accreting.

Apam Beach: This beach (Fig. 3e) has rates of change between 0.9 (accretion) a–d - 4.5 m/yr (erosion). Averagely, the entire beach is eroding at −0.6 m/yr and as noted in Fig. 3, high erosion is observed only at the back beach and close to a small river. This beach is home to a large fishing community that also means many influences from the human activities. Given the erosive nature, it necessitates the implementation of some form of protection.

Glefe Beach: This beach indicated in Fig. 3f was previously a clean sandy and well-known beach for its serene beach line until it developed into a highly eroding site. Our assessment after the coastal protection now shows the side that is protected with a revetment (Table 3, Fig. 3) as having less modifications, with mostly in accretion. The observations however revealed that the extreme western side of the beach remains in erosion. At that side, there is no defense and there is some form of scour introduced at the end of the revetment.

**Fig. 4:**
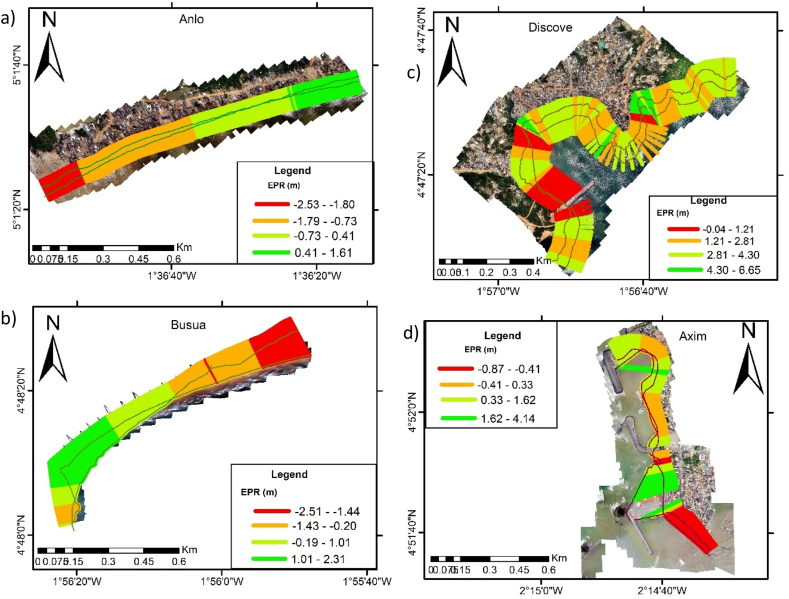
Shoreline changes of beaches along the western coastline a) Anlo Beach; b) Busua Beach, c) Dixcove Beach d) Axim Beach.

Anlo Beach: Anlo beach (Fig. 4a) is a popular open sandy beach known for its annual flooding. It is a quite gently sloping beach within the surfzone. Apart from flooding, erosion is resident on the area possibly because the beach receives less sand from the river Pra. Sediment arriving at this coast through Pra river is believed to be fine because of anthropic activities of gold mining happening upstream. Our results show that the western part of the beach which is at the mouth of the Pra river has the highest amount of erosion. In total, the entire beach erodes at about −0.6 m/yr, peaking at −2.5 m/yr (Fig. 4).

Busua Beach (Fig. 4b): In an earlier study, it was revealed that this beach has been accreting from 2000 to 2020 at an average rate of +0.52 m/yr due to significant influence from tides [46]. Between 2005 and 2022, in the present study, the average change rate is observed as 0, meaning the beach neither retreats inland nor advance seaward. This behaviour is as a result of the beach morphology and sediment discharges from its estuary. To emphasize, this is an embayed shape beach which has restricted movement of sediment and wave induced influence. There is however an indication from our results compared to the [46], that the beach could move into erosion in future if sediment and beach shape change.

Dixcove Beach: Dixcove beach (Fig. 4c) is a complex beach, comprising bays and open shores. Fig. 4 shows the nature of the beach. It was observed that the entire beach is accreting. This beach is accreting, and currently up to about 6.7 m/yr. The rate of accretion is probably because of the embayed nature of the beach, where wave-induced effects are minimal.

Axim Beach: This is also another complex beach (Fig. 4d) but largely defended. Table 3 and Fig. 4 present the range of shoreline changes. EPR results around the defense is showing more stability and less eroded places.

### 3.2 Vulnerability rank of beaches

**Fig. 5:**
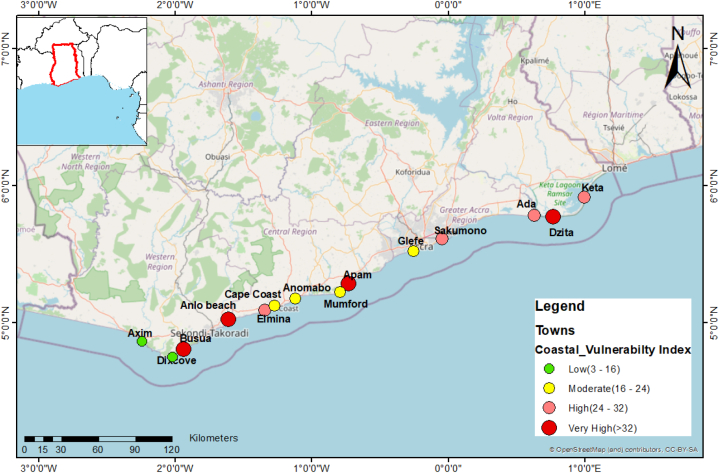
Vulnerability index of the selected beaches along the coastline.

The objective of the coastal vulnerability index (CVI) assessment is to highlight areas where coastal change as a result of coastal hazards and exposure may be more relevant [39] as a quantitative tool to assist in managing resources. Coastal hazards here include erosion, and sea level rise (SLR). In all, the geomorphological and physical parameters (Table 4) used include the tidal regime, geomorphology, the presence of coastal protections (Groynes (G), Revetment®, Jetties (J) and No defense (ND)), erosion rates, SLR and waves. Fig. 5 presents the vulnerability status of the various beaches that were investigated. Most of the undefended beaches (Dzita, Apam, Anlo and Busua) have very high CVIs, meaning they are highly vulnerable to the hazards and exposure factors mentioned here. Six of the defended beaches (Ada, Elmina, Cape Coast, Keta, Sakumono and Glefe) indicate situations of medium to high vulnerability, while the rest of the beaches; Mumford, Dixcove and Axim are of low vulnerable beaches. Table 4 shows all the parameters and the way they were ranked and evaluated to be used for the computation of the CVI (from Equation (2)).

**Table 4:**
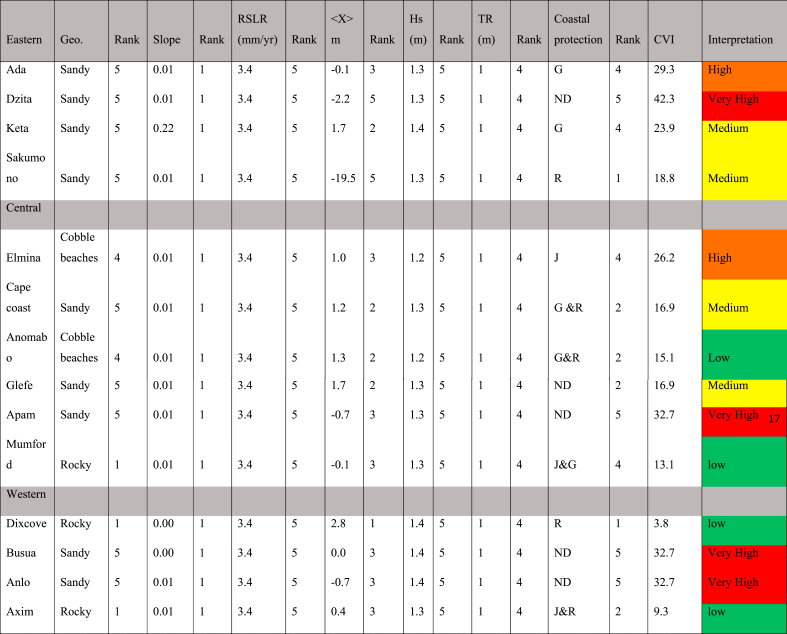
Geomorphological and physical parameters and their ranking.

### 3.3 Knowledge of coastal communities on coastal protection structures

Table 5 summarises the experiences of the investigated communities with coastal protection structures based on the key informants as well as through observations during the field data collections. Even though these interventions are having various negative impacts, for some communities, they are protecting so many other communities and their properties. For instance, at Glefe, the respondents indicated that the design and implementation did not affect them. They indicated the design they have currently was good and were of the hope it would be extended to the other communities that are around Dansoman beach. The respondents submitted that coastal protections prevented floods from the coastal regions and prevented the destruction of houses and the beach by erosion.

**Table 5:** Experience of coastal dwellers with coastal protection structures.

Code	Western	Central	Greater Accra	Volta
Issues	Erosion and Flooding	Erosion and beach degradation	Flooding and sand deposits	Advancement of sea and coastal erosion
Time since changes	30 years	23 years	12 years	30 years
Factors causing coastal erosion	Climate Change	Tidal waves and sand winning	Seasonal changes and climate Changes	Climate change and nature of beach (low elevation)
Coastal Features at Risk	Beach	Beach	Beach and buildings	Beach and buildings
Local coastal protection types	Wood, sandbags, and stones	Sandbags by local community members	Sandbags	Sandbag
Impact on Livelihood	Yes	Yes	No	Yes
Impact on Inhabitants	Loss of fishing assets and fish landing site	Destroyed landing site.	Accumulation of sand	Loss of fishing assets, fish landing sites and demolishing houses
Impact on Community	Beach loss	Loss of coconut trees and fishing assets	None	Houses and land losses
Property losses from communities	Fishing assets and houses	Destruction of houses and landing sites	Personal and company properties	Fishing assets and houses
Benefits of coastal protection strategies	Prevention of floods and beach erosion	Prevents destruction of houses and beach erosion	Prevention of floods	Prevents floodings

## 4 Discussion

### 4.1 Influence of coastal protection on beach stability

We observed that 43% of the fourteen (14) beaches studied showed evidence of erosion while the rest are accreting (building). Of those beaches with protection, 80% are either stable or accreting while 20% are in recession. All the beaches which have no protection are in various stages of erosion. From East to West, coastal protections have been observed to be generally useful in terms of protecting the beach from erosion or flooding. In terms of vulnerability, the beaches of high concern are Dzita, Ada, Sakumono, Glefe, Apam, Anlo and Busua. The study discovered that all the beaches without coastal protection are highly vulnerable which seems to stress the importance of coastal protection structures. For some of these beaches which are located on the downdrift site of the defended shores, their vulnerability is being caused by the hard structures updrift like what has been reported elsewhere (e.g., Ratnayake et al., 2018). For example, Anlo beach downdrift of Shama on the western coast seems to be more vulnerable to both erosion and flooding ever since the sea defense was constructed at Shama; downdrift of Ada Foah seems to experience severe overtopping and erosion as a consequence of the coastal protection structures. However, whiles beaches are being eroded, sediments are transported further away from the eroding beaches thereby accumulating at some other nearby beaches (Amalan et al., 2018).

It can therefore be said that certain areas that had been defended were still vulnerable and subjected to occasional incursions from the sea. Of particular interest are the beaches of Ada and Sakumono which were highly defended including nourishment yet highly vulnerable. This phenomenon confirms the inefficiency of many coastal protections [3]. The nourishment at Ada adopted a uniform design template, with fixed alignments and elevations for both the beach and the dune reinforcement. Sand from a nearby offshore borrow source was hydraulically placed onshore using sinker pipelines [47]. The median grain size diameter of this borrowed material was 540 μm, which was slightly larger compared to the native beach sand [47]. The vulnerability at Ada was largely noticed close to the mouth of the Volta River which seems to influence the dynamics of the beach and subsequently the erosion. It has been reported that a gradual recession of up to 3 m/yr was expected on Ada Beach despite an average equilibrium in the sand balance [48]. This has been attributed to the presence of the Akosombo Dam upstream [20,49] which was done about 60 years ago. Though our observations are aligned with the literature, we further noted that the groyne (G) protection implemented on this beach were constructed at about 700 m apart with cross-shore lengths of 80 m which seems to be inefficient. Larger portions of this protection are in the water presently. These characteristics are different from the ones implemented in Keta (they extended more into the land than in the water). At Glefe and Sakumono, several coastal protections were constructed but erosion rates are still high making these beaches highly vulnerable. Besides, the vulnerability observed around these areas also happens to be contributed by the adjacent areas that the coastal protection does not cover, as in the case of Glefe, Anomabo, and Cape Coast. For most of the areas that are highly vulnerable, erosion and the absence of coastal protection are the predominant factors.

Nonetheless, erosion has also reduced considerably in some areas that have coastal protections. For instance, the beaches at Keta, Mumford, Axim, and Dixcove are now building which were eroding before the construction of the coastal protections. For instance, coastal erosion in Keta was reported at about 4–8 m/yr before the protection, and after the defense, Boateng [20], Boatemaa et al. [50] and Jayson-Quashigah et al. [36] estimated the rates to be 2–4 m/yr, 2.32 m/yr, and 2 m/yr, respectively. Generally, coastal protections would function but their maintenance and extended effect on adjacent beaches has always been challenging [8,19].

### 4.2 Influence of coastal protection on community livelihood

Our results show that most coastal dwellers believe that the structures that are installed on their beaches to stem coastal erosion affect them and their livelihoods. Key areas of socioeconomic lives impacted by the protection include fishing assets such as boats, nets, fishing grounds, and so on. The facilities are either destroyed or lost to structures introduced into their fishing areas. The coastal protection affects their landing sites as well as places for mooring their boats (Table 5). In effect, their fishing livelihoods like fish processing especially relating to the places for drying fish get destroyed. A respondent from Axim complained:

“ …. *it is difficult to berth canoes at the beach area where boulders are laid, the contractor didn’t follow the sea route in the community, and there are stones beneath the sea, which really are a worry to the fishermen, and as such when we are landing at the beach area, we mostly crush into big stones. Often our outboard motors also get destroyed as well, and all these destroy our fishing assets ….“.*

At Dixcove, another respondent noted a similar concern. The effects of coastal protection structures are widespread across the whole coast. In the Volta region, respondents noted that in the construction of the coastal protection, certain houses belonging to the residents were demolished without any compensation to the owners, and this led to the displacement of people in the community. The communities also lost their beaches, vegetation, houses, and land. It was noted that during the preparations for the construction of the Keta coastal protection systems, the communities requested for a fishing harbour, which has since the 1990's not been constructed. Field observations during the study as confirmed by the residents, showed the lack of berthing places and fish landing beaches for the fishers. It was also observed that at certain locations especially in Atorkor in the Anloga district and Lolonyakope in Ada East district, fishing grounds have become very limited as the protection structures provide huge obstacles to the pulling of seine nets during berthing. The presence of these coastal protections therefore in a way resulted in reduced fish catch as a consequence of the difficulty in landing and dragging fishing nets onshore/offshore and thus, affecting their daily occupation or livelihood activities.

For reasons unknown, most communities made it clear that their inputs during the building of such projects were largely not considered. At Dixcove, it was revealed that there was an initial agreement with the contractor to build a safe landing space for the fishermen before the coastal protection, however, they started the construction of the defense first, and that created a challenge for the fisherfolks until a landing beach was finally constructed. At Axim and Keta, the indigenes made suggestions regarding what should be done to avert the negative impact of the defense structure on the communities, but the contractors did otherwise. They consulted with the opinion leaders in the community about 4 times, shared ideas about the defense as well indicating the best approach and design for the community, but the construction company responsible for the construction didn't adhere to the suggestions made by the community leaders.

“.. y*es*, *we met with the authorities about 4 times, but one thing we realized was that, they did not take our suggestions seriously, they felt we were not knowledgeable about the work they were doing in the community, we got troubled a little, they belittled our educational level in the community … … …*”

It is therefore important to also stress the disregard on the side of government, its consultants, and contractors for suggestions from indigenous communities relating to the development of coastal infrastructure. Another important impact of the defense structures on the livelihoods of the communities because of the poor citing of landing beaches is reduction in fish catch. This has forced fish processors to travel to other fishing communities where there are proper landing sites to purchase fish for processing. The cost and risk involved in the travel to purchase fish for processing is therefore enormous. At Anomabo, the community women have to travel to buy fish from cold stores or at beaches where there are Moors to support the safe landing fishing boats. The women suffer a lot, especially those who travel to Elmina or Takoradi harbour to buy fish. Some even travel as far as Tema to buy fish. There may be the need for new policy to ensure safe landing places for fisherfolks, otherwise the works of fishermen will be ruined.

### 4.3 Coastal protection in Ghana

Coastal protection is currently needed now than ever on many parts of the coast but as French [44] would put it, where is coastal defence going? Other questions include what kind of defense is suitable for each unique beach. As many experts put it, some of the solutions, particularly, hard engineering solutions seem to be at war with the environment rather than solving problems [19,48], and one type of solution cannot holistically solve prevent coastal erosion. Nourishment has been combined with groynes at some points like Ada Foah, but the community now complains bitterly about coastal erosion than before.

Along the coastline of Ghana, estimation of the coverage by completed hard-engineered shorelines amounts to about 100 km out of the 550 km, and new ones are being planned and constructed. This extent of the beach was protected in a time space of about 2 decades and by projection, it means the entire coastline could be engineered in a few decades. The implication is huge for the tourism industry, recreational activities and livelihood of coastal communities.

There have been a few cases (e.g., Fuveme, Anlo Beach, etc.) where nature-based solutions like mangrove planting have been tried, though it is challenging to implement such approaches. In Ghana, nature-based solutions [ [8,15]] are less known and the least considered solutions, but if nature-based solutions are expanded along the coastline, they could contribute to economic growth at many sites. The coastal communities should see the need to adopt and implement strategies using nature-based solutions or green infrastructure as alternatives to conventional hardening for erosion protection since that produce minimum losses to riparian and intertidal habitats [ [51,52]].

Community engagement and participation in decision-making processes are critical to ensure that the needs and concerns of local communities are taken into account. Additionally, coastal protection structures should be designed and implemented in a way that minimizes negative impacts on the environment and local livelihoods. Finally, monitoring and evaluation of coastal protection structures are essential to detect failing structures and ensure that they continue to be effective and do not cause unintended negative consequences.

## 5 Conclusion

The present study highlights the shoreline changes in Ghana on highly eroding sites that were either protected by coastal protection or in the process of being defended. Examination of the vulnerability of beaches along hard-engineered coastal protection in Ghana was done from 2005 to 2022. The shoreline change was examined by an End Point Rate method which revealed widespread erosion across the coastline. Most beaches that have been protected experience accretion and those beaches that have no protection are all severely eroding. From East to West, coastal protections have been observed to be generally useful in terms of protecting the beach from erosion or flooding. In terms of vulnerability, the beaches of high concern are Dzita, Ada, Sakumono, Glefe, Apam, Anlo, and Busua. The study discovered that all the beaches without coastal protection are highly vulnerable which seems to stress the importance of coastal protection. In addition to the USGS parameters for the estimation of the coastal vulnerability index, coastal protection presence as a new parameter revealed their importance in reducing the vulnerability of defended coasts. The results show that most parts of the coast are now vulnerable in contrast to what was previously observed that only the eastern part of the coast was vulnerable. Apart from the ‘knock-on effect’ that coastal protections cause to other beaches, the construction of hard-engineered coastal protection such as groynes and revetments significantly affects the livelihood of community members because the suggestions of community members are hardly accepted in decision making. Grey coastal protection decreases livelihood opportunities for coastal communities.

This paper revealed that the manner of implementation of the coastal projection structures produced more chances of erosion and effects on communities. The study also revealed that the application of nature-based solutions in Ghana and possibly many other coastal countries is low. It is proposed that during the implementation of these protections, community leaders should be consulted, and their suggestions considered.

## Data availability statement

Data is being archived in google drive (https://drive.google.com/drive/folders/1oTvRJvIpq9DRlSmBUHJb4nZZ5Tnw8RKx) and will be made available on request.

## CRediT authorship contribution statement

**Bapentire Donatus Angnuureng:** Conceptualization, Formal analysis, Funding acquisition, Methodology, Project administration, Visualization, Writing – original draft, Writing – review & editing. **Richard Adade:** Data curation, Investigation, Writing – review & editing. **Ernest Obeng Chuku:** Conceptualization, Methodology, Writing – review & editing. **Selorm Dzantor:** Data curation, Writing – review & editing. **Emmanuel Kwadwo Brempong:** Data curation, Formal analysis, Writing – review & editing. **Precious Agbeko Dzorgbe Mattah:** Project administration, Supervision, Writing – review & editing.

## Declaration of competing interest

The authors declare that they have no known competing financial interests or personal relationships that could have appeared to influence the work reported in this paper.
